# Serum biomarkers and bone mineral density of the hip in Chinese elderly patients with hip fractures

**DOI:** 10.1097/MD.0000000000042954

**Published:** 2025-06-20

**Authors:** Luhui Liu, Shuang Chen, Qiang Huang

**Affiliations:** a Department of Orthopedic Surgery, The People’s Hospital of Sixian County, Anhui Province, China; b Department of Orthopaedics and Traumatology, Beijing Jishuitan Hospital, Capital Medical University, Beijing, China.

**Keywords:** bone mineral density, hip fracture types, serum biomarkers

## Abstract

This study aims to analyze the serum biomarkers and bone mineral density (BMD) of the hip in patients with hip fractures, and to explore the risk factors for different types of hip fractures in Chinese elderly patients. In this retrospective case-control study, 235 patients aged >60 and suffered from their first hip fracture were included. The patients were divided into 2 groups: the femoral neck fracture group (n = 99, mean age 80.6 ± 7.76 yr) and the intertrochanteric fracture group (n = 136, mean age 83.5 ± 6.82 yr). Serum biomarkers and BMD parameters (femoral neck, trochanter, intertrochanteric region, total hip, and Ward triangle) of the contralateral hip were collected and compared.The intertrochanteric fracture group had significantly lower red blood cell count, hemoglobin, albumin, total cholesterol, and serum calcium levels than the femoral neck fracture group (*P* < .001), but had higher blood urea nitrogen levels (*P* = .01). In both male and female patients, the intertrochanteric fracture group had significantly lower BMD in the greater trochanter and total hip regions compared to the femoral neck fracture group (*P* < .05). Logistic regression analysis showed that advanced age, lower hemoglobin, and lower total cholesterol levels were significant risk factors for intertrochanteric fractures (odds ratio [OR] = 1.06, *P* = .016; OR = 3.65, *P* < .001; OR = 3.20, *P* = .017), while higher greater trochanter BMD was a protective factor (OR = 0.36, *P* < .001).Different risk factors are associated with femoral neck and intertrochanteric fractures. Some of the serum biomarkers and hip BMD parameters are closely related to the types of fractures.

## 1. Introduction

With the growing aging population in China, the number of patients suffering from hip fractures has been increasing annually.^[1,2]^ Prevention of hip fractures has therefore become increasingly important. Hip fractures are primarily categorized into femoral neck and intertrochanteric fractures based on the fracture location. Previous research has suggested that patients with intertrochanteric fractures have more severe osteoporosis.^[3]^ Additionally, for non-simultaneous bilateral hip fractures, the concordance rate for fracture type is reported to be 72 to 80%,^[4,5]^ suggesting differences in the underlying mechanisms of these 2 fracture types. This study collected serum biomarkers and dual-energy X-ray absorptiometry (DXA)-measured bone mineral density (BMD) values from hip fracture patients to investigate the risk factors for different types of hip fractures.

## 2. Patients and methods

### 2.1. Study participants

The inclusion criteria was patients sustained their fractures from low-energy falls. The exclusion criteria were patients with subtrochanteric fractures, high-energy injuries, pathological fractures, previous osteonecrosis of the femoral head, chronic kidney disease, liver dysfunction, gastrointestinal absorption disorders, thyroid diseases, chronic alcohol use, or those taking medications affecting bone metabolism.

This study retrospectively enrolled 235 patients aged >60 who suffered from their first osteoporotic hip fracture and were treated in our hospital between January 2020 and January 2024. The patients were divided into 2 groups: the femoral neck fracture group (n = 99, 24 males, mean age 80.6 ± 7.76 yr, body weight 66.1 ± 11.3 kg, body mass index [BMI] 22.2 ± 3.42 kg/m²), and the intertrochanteric fracture group (n = 136, 35 males, mean age 83.5 ± 6.82 yr, body weight 64.9 ± 12.9 kg, BMI 21.9 ± 4.03 kg/m²).

### 2.2. Serum biomarker testing

Serum biomarkers were measured within 12 hours of admission using standardized automatic laboratory equipment. The biomarkers included blood cell count, hemoglobin, albumin, total cholesterol, serum calcium, and blood urea nitrogen levels.

### 2.3. BMD testing (DXA)

DXA scans of the contralateral hip were performed using X-ray bone densitometer (Discovery A, Hologic, Marlborough). BMD values (g/cm²) were measured in 5 hip subregions: femoral neck, greater trochanter, intertrochanteric region, whole region of the hip, and Ward triangle.

### 2.4. Statistical analysis

SPSS 22.0 software (IBM, Armonk) was used for statistical analysis. Continuous variables were expressed as mean ± SD. ANCOVA was used to compare continuous variables between the 2 groups, adjusting for age and BMI. Pearson partial correlation analysis was used to determine the relationship between serum biomarkers and BMD. Logistic regression analysis was used to identify risk factors for each fracture type. A *P* value < .05 was considered statistically significant.

## 3. Results

### 3.1. Serum biomarkers and fracture types

Patients in the intertrochanteric fracture group had significantly lower red blood cell counts, hemoglobin, albumin, total cholesterol, and serum calcium levels compared to the femoral neck fracture group (*P* < .001), but higher blood urea nitrogen levels (*P* = .01) (Table 1).

**Table 1:** Comparison of serum markers between the 2 hip fracture groups after adjustment of age.

Serum markers	Femoral neck group (n = 99)	Intertrochanteric group (n = 136)	*F* value	*P* value
Neutrophil count (10^9^/L)	7.65 ± 2.31	7.45 ± 2.50	0.814	.368
Red blood cell (10^12^/L)	4.05 ± 0.51	3.44 ± 0.61	55.36	**<.001**
Hemoglobin (g/L)	119.8 ± 13.59	102.6 ± 17.02	57.95	**<.001**
Albumin (g/L)	38.5 ± 4.01	36.4 ± 4.51	11.69	**<.001**
Urea nitrogen (mmol/L)	6.12 ± 2.25	7.21 ± 2.67	6.725	**.010**
Creatinine (mmol/L)	70.2 ± 24.00	71.7 ± 23.42	0.229	.633
Total cholesterol (mmol/L)	4.39 ± 1.03	3.83 ± 0.80	20.21	**<.001**
Triglyceride (mmol/L)	0.94 ± 0.40	1.02 ± 0.50	0.670	.503
Calcium (mmol/L)	2.13 ± 0.11	2.07 ± 0.11	14.42	**<.001**
Phosphate (mmol/L)	1.02 ± 0.20	1.07 ± 0.82	0.318	.573
Magnesium (mmol/L)	0.85 ± 0.09	0.84 ± 0.10	0.569	.487

Bold values indicate *P* < .05.

### 3.2. BMD parameters and fracture types

In male patients, the intertrochanteric fracture group had significantly lower BMD in the greater trochanter and the whole hip region compared to the femoral neck fracture group (*P* = .041 and *P* = .031, respectively). In female patients, BMD in the greater trochanter, total hip, and Ward triangle was significantly lower in the intertrochanteric fracture group (*P* < .05), while no significant difference was observed in femoral neck and intertrochanteric region BMD between the 2 groups (*P* > .05) (Table 2).

**Table 2:** Comparison of bone mineral density parameters between the 2 hip fracture groups after adjustment of age, weight, and BMI.

BMD parameters (g/cm^2^)	Femoral neck group	Intertrochanteric group	*F* value	*P* value
Male patients	n = 24	n = 35		
Femoral neck	0.530 ± 0.048	0.503 ± 0.053	2.872	.108
Greater trochanter	0.497 ± 0.068	0.461 ± 0.055	4.406	**.041**
Intertrochanteric	0.845 ± 0.114	0.778 ± 0.108	3.847	.055
Whole hip region	0.695 ± 0.074	0.646 ± 0.078	4.899	**.031**
Ward triangle	0.343 ± 0.094	0.307 ± 0.072	2.330	.133
Female patients	n = 75	n = 101		
Femoral neck	0.461 ± 0.067	0.438 ± 0.085	0.736	.392
Greater trochanter	0.451 ± 0.060	0.403 ± 0.075	18.370	**<.001**
Intertrochanteric	0.698 ± 0.121	0.650 ± 0.145	2.254	.135
Whole hip region	0.587 ± 0.084	0.546 ± 0.108	4.193	**.042**
Ward triangle	0.304 ± 0.078	0.266 ± 0.085	5.390	**.021**

Bold values indicate *P* < .05.

BMD = bone mineral density, BMI = body mass index.

### 3.3. Correlation between serum biomarkers and BMD

After adjusting for age, weight, and BMI, hemoglobin levels in the femoral neck fracture group were negatively correlated with femoral neck BMD (*r* = −0.211, *P* = .03), and total cholesterol levels were negatively correlated with intertrochanteric region BMD (*r* = −0.249, *P* = .014). In the intertrochanteric fracture group, hemoglobin levels were positively correlated with greater trochanter, intertrochanteric region, and whole hip region BMD (*P* < .05), while albumin levels were positively correlated with greater trochanter BMD (*R* = 0.191, *P* = .028) (Table 3).

**Table 3:** Partial correlative analysis of serum markers with statistical difference and BMD parameters.

Serum parameters	Femoral neck		Greater trochanter		Intertrochanteric		Whole hip region		Ward triangle	
	*r*	*P*	*r*	*P*	*r*	*P*	*r*	*P*	*r*	*P*
Femoral neck group (n = 99)										
Hemoglobin	−0.211†	.030	0.096	.354	−0.096	.350	−0.054	.604	0.056	.586
Albumin	−0.116	.259	−0.008	.937	−0.149	.146	−0.121	.241	−0.046	.658
Urea nitrogen	0.072	.487	0.153	.136	0.112	.279	0.125	.224	−0.094	.362
Total cholesterol	−0.253	.013	−0.005	.961	−0.249*	.014	−0.232	.023	−0.113	.271
Calcium	−0.091	.378	−0.008	.940	−0.155	.131	−0.093	.370	−0.073	.480
Intertrochanteric group (n = 136)										
Hemoglobin	0.167	.054	0.266†	.002	0.204*	.019	0.226†	.009	0.135	.121
Albumin	0.063	.472	0.191*	.028	0.135	.122	0.139	.110	−0.023	.794
Urea nitrogen	0.061	.486	−0.088	.316	0.048	.583	0.017	.850	−0.037	.675
Total cholesterol	−0.158	.069	−0.070	.423	−0.036	.684	−0.057	.518	−0.171	.085
Calcium	0.045	.611	0.024	.785	−0.085	.332	−0.061	.489	0.041	.639

BMD = bone mineral density.

* Correlation is significant at the 0.05 level (2-tailed).

† Correlation is significant at the 0.01 level (2-tailed).

### 3.4. Risk factors for fracture types

Logistic regression analysis revealed that advanced age, lower hemoglobin, and lower total cholesterol levels were significant risk factors for intertrochanteric fractures (odds ratio [OR] = 1.06, *P* = .016; OR = 3.65, *P* < .001; OR = 3.20, *P* = .017). Higher greater trochanter BMD was a protective factor (OR = 0.36, *P* < .001) (Table 4).

**Table 4:** Risk factors for intertrochanteric fractures compared with femoral neck fracture by logistic regression.

Variables	Odds ratio (OR)	95% confidence interval (95% CI)	*P* value
Age	1.06	1.02–1.09	.016
Hemoglobin	3.65	2.14–6.23	<.001
Total cholesterol	3.20	1.23–8.29	.017
BMD of greater trochanter	0.36	0.21–0.63	<.001

BMD = bone mineral density.

## 4. Discussion

This study reveals significant differences in serum biomarkers and BMD between patients with femoral neck fractures and those with intertrochanteric fractures. Previous research has reported associations between different types of hip fractures and serum biomarkers. For instance, Fisher et al^[6]^ found that patients with intertrochanteric fractures had significantly lower levels of hemoglobin and albumin compared to those with femoral neck fractures. Similarly, Sari et al^[7]^ reported that patients with intertrochanteric fractures had notably lower hemoglobin and serum calcium levels. Our findings align with these studies, showing that hemoglobin, albumin, and serum calcium levels are lower in patients with intertrochanteric fractures. Elderly individuals often experience reduced nutritional intake, compounded by chronic conditions such as hypertension and diabetes, leading to a higher incidence of malnutrition.^[8]^ The lower levels of hemoglobin and albumin in intertrochanteric fracture patients suggest poorer nutritional status.

Additionally, the lower serum calcium levels observed in intertrochanteric fracture patients may be related to lower vitamin D levels^[9]^ and higher parathyroid hormone levels.^[10]^This study also found lower total cholesterol and higher blood urea nitrogen levels in intertrochanteric fracture patients. Atac et al^[11]^ reported a positive correlation between total cholesterol levels and hemoglobin levels, which is consistent with the lower total cholesterol observed in our intertrochanteric fracture group. With advancing age, decreased liver and kidney function impairs the excretion of metabolic waste products, which may explain the higher blood urea nitrogen levels and poorer kidney function in intertrochanteric fracture patients compared to those with femoral neck fractures. Fox et al^[12]^ found that poorer overall health is an independent risk factor for intertrochanteric fractures.

Many studies have used BMD measurements to investigate the relationship between hip BMD parameters and fracture types. Wu et al^[13]^ found that intertrochanteric fracture patients had significantly lower BMD in the greater trochanter compared to femoral neck fracture patients. Yongun et al^[14]^ and Kanazawa et al^[15]^ reported that both greater trochanter and intertrochanteric BMD were significantly lower in intertrochanteric fracture patients. Our study found significantly lower BMD in the greater trochanter and whole hip region in both male and female patients with intertrochanteric fractures. Higher BMD in the proximal femur, including the greater trochanter and intertrochanteric regions, indicates greater trabecular bone strength, which helps maintain bone integrity. Consequently, higher BMD in these regions may lead to femoral neck fractures when the force is transferred to the less resilient femoral neck and Ward triangle regions.^[15–17]^ Logistic regression analysis confirmed that lower BMD in the greater trochanter is a risk factor for intertrochanteric fractures. The severity of osteoporosis may also have predictive value for hip fracture risk, which needs further investigation.

Previous studies have focused solely on differences in serum biomarkers or BMD between fracture types, with limited research combining both aspects. Japanese researchers have reported that lower serum albumin levels are associated with lower BMD.^[18]^ Our study, which examined the relationship between serum biomarkers and BMD in 5 hip subregions, found that after adjusting for age, weight, and BMI, hemoglobin levels were positively correlated with BMD in the greater trochanter, intertrochanteric region, and total hip in intertrochanteric fracture patients, while albumin levels were positively correlated only with greater trochanter BMD. These findings suggest that specific serum biomarkers and BMD parameters are closely related to the fracture types. Lower hemoglobin and albumin levels in elderly patients are associated with lower BMD in the intertrochanteric regions, which increases the risk of intertrochanteric fractures during falls. This study also identified different risk factors for femoral neck and intertrochanteric fractures. These results suggest that targeted prevention strategies, such as correcting anemia and improving nutritional status to enhance proximal femur BMD, could help reduce the incidence of intertrochanteric fractures.

However, this study has some limitations. Although serum biomarkers were collected within 24 hours of fracture, they may not accurately reflect pre-fracture levels. Intertrochanteric fractures, being extracapsular, often have higher associated bleeding than femoral neck fractures due to the lack of surrounding capsule. Thus, differences in hemoglobin levels may be influenced by varying amounts of bleeding, which introduces some error. In future studies, we aim to incorporate trabecular bone index, 25(OH)D and other parameters for a more comprehensive assessment of osteoporosis. Additionally, the relatively small sample size necessitates further validation through larger studies.

## Author contributions

**Conceptualization:** Luhui Liu, Shuang Chen, Qiang Huang.

**Data curation:** Luhui Liu, Shuang Chen, Qiang Huang.

**Formal analysis:** Luhui Liu, Shuang Chen, Qiang Huang.

**Investigation:** Luhui Liu, Shuang Chen, Qiang Huang.

**Methodology:** Luhui Liu, Shuang Chen, Qiang Huang.

**Project administration:** Luhui Liu, Shuang Chen, Qiang Huang.

**Resources:** Qiang Huang.

**Software:** Qiang Huang.

**Supervision:** Qiang Huang.

**Validation:** Shuang Chen, Qiang Huang.

**Visualization:** Shuang Chen, Qiang Huang.

**Writing – original draft:** Luhui Liu, Shuang Chen, Qiang Huang.

**Writing – review & editing:** Qiang Huang.
